# *Lactobacillus reuteri* strain 8008 attenuated the aggravation of depressive-like behavior induced by CUMS in high-fat diet-fed mice through regulating the gut microbiota

**DOI:** 10.3389/fphar.2023.1149185

**Published:** 2023-03-27

**Authors:** Canye Li, Zuanjun Su, Zhicong Chen, Jinming Cao, Xiufeng Liu, Feng Xu

**Affiliations:** ^1^ Fengxian Hospital, Southern Medical University, Shanghai, China; ^2^ Sixth People’s Hospital South Campus, Shanghai Jiaotong University, Shanghai, China

**Keywords:** chronic unpredictable mild stress, obesity, depression, *Lactobacillus* reuteri, mice

## Abstract

**Objective:** Gut microbiota play a key role in the pathogenesis of obesity and depression. Probiotics are a preventive strategy for obesity and a novel treatment for depression symptoms. However, the ameliorative or therapeutic effect of potential probiotic candidate *Lactobacillus reuteri* (*L. reuteri*) on obesity and depression comorbidity still remains unclear. We investigated the effects of chronic unpredictable mild stress (CUMS) in high-fat diet-fed mice and the effects of *Lactobacillus reuteri* strain 8008 on various disease indicators of obesity and depression comorbidity disease.

**Methods:** Forty male C57BL/6 mice were randomized into 2 groups: the normal control (NC) group (*n* = 10) and the high-fat diet (HFD) group (*n* = 30), being fed with normal diet (ND) or high-fat diet (HFD) for 8 weeks, respectively. Then the obese mice fed with HFD were randomly allocated into 3 sub-groups: the HFD group (*n* = 10); the HFD + CUMS group (*n* = 10); the HFD + CUMS + L.r group (*n* = 10). The latter 2 subgroups underwent CUMS for 4 weeks to build the obesity and depression comorbidity mice model. During the duration of treatment, mice were gavaged with 0.5 mL PBS solution or *L. reuteri* (2 × 10^9^ CFU/mL) once a day, respectively. The body weight, food intake, organ weight, behavioral indicators, histology, blood lipids, levels of inflammatory cytokines and tight junction proteins and abundance of colonic contents bacteria were measured.

**Results:** The obesity and depression comorbidity mice model was successfully established after HFD feeding and chronic stress. The comorbid mice demonstrated inflammatory responses increase in liver and adipose tissues, worsened damage to the intestinal barrier as well as gut microbiota disorder. Gavaged with *L. reuteri* attenuated depressive-like behavior, improved blood lipids and insulin resistance, reduced inflammation in liver and adipose tissues, improved intestinal tight junctions as well as the microbiome dysbiosis in obesity and depression comorbidity mice.

**Conclusion:** *Lactobacillus reuteri* strain 8008 could alleviate depressive-like behaviors and related indicators of obesity disorders by regulating the gut microbiota in obesity and depression comorbid mice.

## Introduction

The availability of low-cost manufactured high-fat foods has boosted the worldwide spread of obesity, which has developed into one of the greatest risk factors for metabolic diseases (Ford et al., 2017). Obesity is defined as a state of being significantly overweight and having excessive layers of fat, resulting from the accumulation of excessive fats, especially triglycerides, in the body. The World Health Organization (WHO) reported by 2022 that the number of obese people worldwide had exceeded one billion, of whom 650 million were adults, 340 million were adolescents and 39 million were children (WHO, 2022). Obesity not only increases the risk of cardiovascular diseases and diabetes, but also leads to depression and other psychological diseases comorbidity (Conway and Rene, 2004; Piché et al., 2020). Obese patients had an increased risk (∼55%) of developing depression in their lifetime. Obese patients tend to suffer from social stress such as verbal or physical abuse secondary to being overweight or obese, and these stresses exposure may increase the risk of developing depression (Fettich and Chen, 2012; Wu and Berry, 2018).

Clinical evidence shows that depression disorder is the most common mental disorder that co-occurs in obese patients (Wang et al., 2019). Patients with depression tend to exhibit slowness of thought, agitation and sustained low mood, even suicidal tendency (Zhuang et al., 2018). Depressed patients also increased obesity risk (∼58%) (de Wit et al., 2010; Luppino et al., 2010; Bergantin, 2020). Although there is a strong linkage between the obesity and depression, the exact mechanisms of the reciprocal interactions are still unclear.

Gut microbes play a key role in the pathogenesis of diseases including psychiatric and metabolic disorders. Alterations in host-microbiota and reductions in bacterial diversity were linked to both obesity and depression (Lynch and Pedersen, 2016; Zmora et al., 2019; Simpson et al., 2021). The ratio of the *Bacteroidetes* and *Firmicutes*, two leading phylum classes of the intestinal flora, in obesity-depression comorbidity patients is different from normal subjects (Gomes et al., 2018; Stanislawski et al., 2019; Simpson et al., 2021). Probiotics are a preventive strategy for obesity and a novel treatment for depression symptoms (Kim et al., 2022; Maybee et al., 2022). Probiotics might restore the integrity of the mucosal barrier, improve inflammation and promote metabolic homeostasis in obesity patients by influencing the composition of the intestinal flora (Wrzosek et al., 2013). Moreover, probiotics could alleviate depressive-like behavior in depressed animal models (Hao et al., 2019). These suggested that probiotics might have a potential ameliorative or therapeutic effect on obesity and depression comorbidity.

*Lactobacillus reuteri* (*L. reuteri*), also called *Limosilactobacillus reuteri*, is a lactic acid bacteria existing in the intestinal tract of all vertebrates and mammals, which can resist the attack of stomach acid and adhere strongly to the intestinal mucous membrane. It can also improve the distribution of intestinal flora, antagonize the colonization of harmful bacteria and prevent intestinal diseases. *Lactobacillus reuteri* extensively inhibits the growth of Gram-positive and Gram-negative bacteria, Yeasts, Fungus and pathogens (Dargenio et al., 2021). It has been reported to prevent hyperlipidemia caused by HFD by facilitating cholesterol and bile salt excretion and maintaining intestinal mucosal barrier function, and also has a preventive effect on stress-induced anxiety/depression. These indicated that *L. reuteri* might have therapeutic value in treating obesity and depression comorbidity (Jones et al., 2012; Li S. et al., 2019; Jang et al., 2019). However, the potential therapeutic mechanisms of *L. reuteri* in obesity and depression comorbidity are still not clear. In this study, we built the obesity and depression comorbid mice model to investigate the potential ameliorative or therapeutic effects and possible mechanisms of *Lactobacillus reuteri* strain 8008.

## Materials and methods

### Bacterial strains and growth conditions

*Lactobacillus reuteri* strain 8008 used in this study was obtained from BeNa Culture Collection (BNCC 337178). The microorganism was cultured in the MRS broth medium (M8540, Solarbio, Beijing, China) at 37°C, 250 rpm for 16 h. The cultures were then centrifuged at 4°C, 3,000 rpm for 10 min, and washed with sterile phosphate buffer saline (PBS, pH 7.2) 3 times. The concentration of bacterial fluid was measured with a bacterial turbidimeter (WGZ-XT, Hangzhou Qiwei Instrument Co., Ltd.). The final concentration was adjusted to 2 × 10^9^ colony-forming units (CFU)/ml with PBS. The experiments described above were performed under strictly micro-aerobic conditions.

### Animals

Male C57BL/6 mice (28–30 g) of about 5 months were bought from Jiangsu Huachuang Pharma Tech Co., Ltd. (License#: SCXK 2020-0009). All mice were acclimatized for 1 week in a specific pathogen-free (SPF) laboratory at East China Normal University, Shanghai, under a 12-h diurnal cycle at 21°C–25°C with relative humidity between 50% and 60%. All animals had free access to water and food.

### Obesity and depression comorbidity model establishment and experimental design

After 1 week of adaptation, mice were randomized into two groups: normal control (NC) group fed with a normal diet (ND:10% fat, 70% carbohydrate, and 20% protein); high-fat diet (HFD) group fed with a high-fat diet (HFD: 60% fat, 20% carbohydrate, and 20% protein). After 8 weeks of feeding, the obesity was formed and the weights of mice in the HFD group (*n* = 30) were 30% higher than those in the NC group (*n* = 10).

**FIGURE 1:**
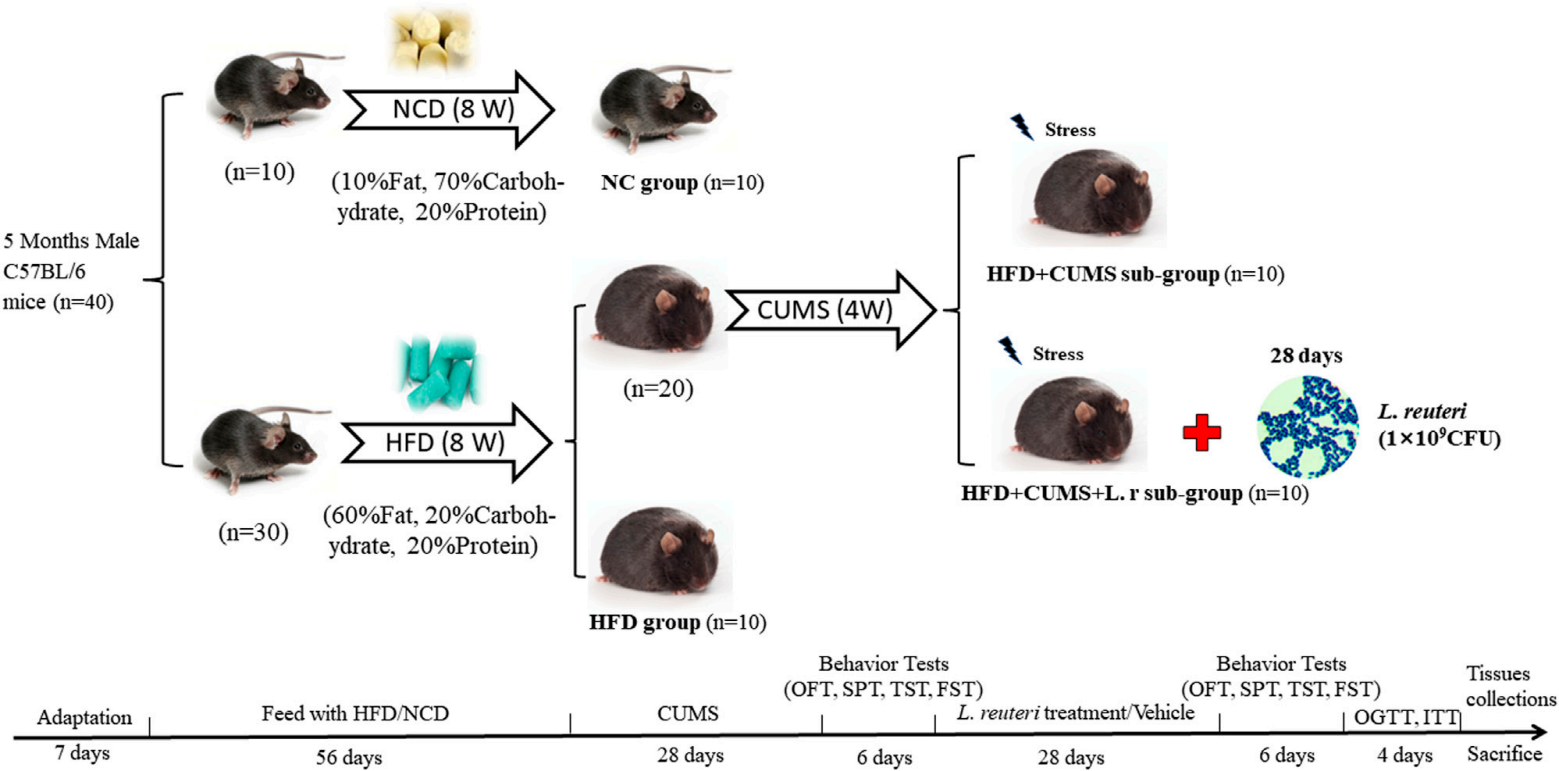
Scheme of the animal experimental design. All mice were adapted for 1 week before the experiments were conducted. Then mice were randomly divided into two groups (NC group, fed a normal diet, *n* = 10; HFD group, fed a high-fat diet, *n* = 30). After 8 weeks of feeding, the HFD group was randomly divided into three subgroups. (HFD group, *n* = 10; HFD + CUMS group, *n* = 10; HFD + CUMS + L.r group, *n* = 10). The HFD + CUMS and the HFD + CUMS + L.r sub-groups underwent 4-week chronic unpredictable mild stress (CUMS) interventions. Followed by 6 days of behavior tests (behavior tests in the order of OFT, SPT, TST and FST). After behavior tests, the HFD + CUMS + L.r group underwent a 4-week gavage of 0.5 mL *Lactobacillus reuteri* (2 × 10^9^ CFU/mL) and the other groups underwent an equal dose of PBS. Then behavior tests were performed for 6 days after the end of the gavage. Later mice were performed the OGTT and ITT. Finally, mice were sacrificed and the tissue samples were collected.

Then the 30 obese mice in HFD group were randomly allocated into 3 sub-groups: the HFD group (*n* = 10); the HFD + CUMS group (*n* = 10); the HFD + CUMS + L.r group (*n* = 10). In the following 4 weeks, the NC and HFD groups were not given any interventions, and the HFD + CUMS and HFD + CUMS + L.r groups underwent chronic unpredictable mild stress (CUMS) to exacerbate depression-behavior phenotype. In the duration of treatment, mice in the NC, HFD and HFD + CUMS groups were gavage with 0.5 mL PBS solution and the HFD + CUMS + L.r group was gavage with 0.5 mL *L reuteri* solution (2 × 10^9^ CFU/mL) once a day, respectively. The mice were housed collectively (5 mice/cage) in the NC and HFD groups and housed individually (1 mouse/cage) in the HFD + CUMS and HFD + CUMS + L.r sub-group. The detailed experimental design flow was shown in Figure 1.

### CUMS procedure

The CUMS procedure was performed as previously described (Nollet, 2021), with slight modification. In short, the CUMS protocol contained a variety of mild stressors: ice water swimming (4°C, 5 min), noise stimulation (3 h), body restriction (3 h), tilted cage (24 h), cage shaking (15 min), damp bedding (24 h), empty cage (24 h) and turned night into day (24 h). Mice were submitted daily to two different stressors for 4 weeks. The details of the interventions performed were shown in Table 1.

**TABLE 1:** Chronic unpredictable mild stress (CUMS) processes.

Stressors	Day of CUMS procedures												
Ice water swimming	3	8	12	16	21								
Noise stimulation	3	5	6	10	12	14	18	22					
Body restriction	1	2	7	9	11	15	17	20	22	24	25	28	
Tilted cage	2	4	8	10	13	18	19	23	26				
Cage shaking	1	7	11	15	17	24	25	27					
Damp bedding	5	14	19	26	27								
Empty cage	6	13	20	21									
Turned night into day	4	9	16	23	28								

### Behavioral testing

The behavioral tests were conducted on consecutive 6 days in orders as open field test (OFT), sucrose preference test (SPT), tail suspension test (TST) and forced swimming test (FST). The time interval between two consecutive behavioral tests was 24 h.

### Open field test (OFT)

Mice were acclimatized in a quiet room for 30–60 min prior to the test. The open field test apparatus consisted of an opaque plastic box (100 cm × 100 cm × 40 cm) divided equally into 20 cm^2^ × 20 cm^2^ squares. Mice were placed individually at the center of each box and their spontaneous activities were recorded by using the Tracking Master V3.0 software (ZSDC Science and Technology Development Co., Ltd.) for 5 min. The animals should be stroked for 1–2 min daily to reduce the impact of non-specific stress stimulation on animals before experiments.

### Sucrose preference test (SPT)

Mice were housed in single cages and trained to drink sucrose solution for 48 h (1% sucrose solution and water were given throughout the training session, exchanging two bottle positions every 24 h). After training the mice were deprived of water for 12 h and then given 1% sucrose solution and water. The amount of water (g) drank by mice from two bottles was measured after 12 h (exchanging the positions of two bottles once during the period). The following equation was used to calculate the sucrose preference: sucrose preference index (%) = (sucrose solution consumed/total solution consumed) × 100%.

### Tail suspension test (TST)

Mice were suspended by the tail in an upside-down position (3/4 of the tail was fixed to a hook) and the head should be kept at a certain distance (about 30 cm) from the bottom of the suspension box and the experiment was conducted for 6 min. The immobility time was recorded by using the Tracking Master V3.0 software (ZSDC Science and Technology Development Co., Ltd.) for the last 5 min.

### Forced swimming test (FST)

Mice were gently placed in a circular container (30 cm diameter) filled with 22°C water with a depth of 20 cm. The swimming test was performed for 6 min and the immobility times were recorded by using the Tracking Master V3.0 software (ZSDC Science and Technology Development Co., Ltd.) for the last 5 min.

### Blood sampling and tissue extraction

After fasting for 12 h, mice were anaesthetized, then the eyeballs were removed to collect blood through the sockets. The blood samples were placed at room temperature for 2 h and centrifuged 3,000 rpm at 4°C for 15 min. The serum was collected and stored at −80°C. The brains, livers, colons, white adipose tissues (epididymis fat and perinephric fat) and colon contents of mice were collected and temporarily stored in liquid nitrogen, then subsequently stored at −80°C.

### Blood glucose, serum insulin, oral glucose tolerance/insulin tolerance test, and HOMA-IR index

The end of tail of mice was cut off by 1–2 mm with scissors and the tail was gently squeezed to collect the blood into a drop, then the fasting blood glucose of mice were measured by blood glucose test strips. Serum insulin was quantified by enzyme-linked immunosorbent assay (ELISA) kits (Proteintech Group, United States) according to the manufacturer’s instructions. The oral glucose tolerance and insulin tolerance test were conducted with reference to literature (Andrikopoulos et al., 2008). For the OGTT, mice oral gavage with glucose solution (2.0 g/kg). For the ITT, mice were intraperitoneally injected with recombinant human insulin (0.5 IU/kg) 48 h after the end of the OGTT. After the mice fasted for 5 h, the end of the mice tails was cut off by 1–2 mm with scissors, and blood from tails was collected to detect blood glucose levels 30 min before the administration of glucose and injection of insulin and 0, 15, 30, 60, 90, and 120 min afterwards. The following equation was used to calculate the homeostasis model assessment of the IR (HOMA-IR) index: [fasting blood glucose levels (mmol/L)] × [fasting serum insulin levels (mIU/L)]/22.5.

### Lipid analysis

Serum lipids including total cholesterol (TC), triacylglycerol (TG), non-esterified fatty acid (NEFA), high-density lipoprotein cholesterol (HDL-C), and low-density lipoprotein cholesterol (LDL-C) levels were quantified using the Beckman Coulter AU5800 Automatic Biochemistry Analyzer (Beckman Coulter, Shanghai, China)**.**

### Histopathological examination

The tissue samples (4–5 μm in thickness) were submerged in 10% formalin solution. The H&E, AB-PAS and Oil Red O staining were used to stain the tissue sections. The size of adipocyte cells, goblet cell quantity, colon intestinal villus height, intestinal wall thickness, and intestinal mucus thickness were measured by using ImageJ software.

### Determination of mRNA expression

Total RNA of colon, livers and white adipose tissues were extracted with TRIzol (Invitrogen™, Carlsbad, United States). The extracted RNA was reverse transcribed to complementary DNA (cDNA) by using Evo M-MLV RT Mix Kit (ACCURATEBIOTECHNOLOGY(HUNAN) CO, LTD., Changsha, China). For qRT-PCR, SYBR Green Premix Pro Taq HS qPCR Kit (ACCURATEBIOTECHNOLOGY(HUNAN) CO, LTD., Changsha, China) was used according to the manufacturer’s protocol. Relative gene expression was done using the 2^−ΔΔCT^ method. The list of primers was shown in Table 2.

**TABLE 2:** The primers sequences.

Primer	Forward primer	Reverse primer	Product size (bp)
IL-6	TAG​TCC​TTC​CTA​CCC​CAA​TTT​CC	TTG​GTC​CTT​AGC​CAC​TCC​TTC	76
IL-10	GCT​GGA​CAA​CAT​ACT​GCT​AAC​C	ATT​TCC​GAT​AAG​GCT​TGG​CAA	78
IL-1β	GAA​ATG​CCA​CCT​TTT​GAC​AGT​G	TGG​ATG​CTC​TCA​TCA​GGA​CAG	116
TNF-α	CCT​GTA​GCC​CAC​GTC​GTA​G	GGG​AGT​AGA​CAA​GGT​ACA​ACC​C	148
ZO-1	GCT​TTA​GCG​AAC​AGA​AGG​AGC	TTC​ATT​TTT​CCG​AGA​CTT​CAC​CA	156
Occludin	TGA​AAG​TCC​ACC​TCC​TTA​CAG​A	CCG​GAT​AAA​AAG​AGT​ACG​CTG​G	128
Claudin-1	GGA​CAG​GAG​CAG​GAA​AGT​AGG​A	CCC​ATC​AAT​GCC​AGG​TAT​GAA	101
Claudin-2	CAA​CTG​GTG​GGC​TAC​ATC​CTA	CCC​TTG​GAA​AAG​CCA​ACC​G	128
KLF4	GTT​GAC​TTT​GGG​GCT​CAG​GTA	CAT​GTC​AGA​CTC​GCC​AGG​T	100
GAPDH	AGG​TCG​GTG​TGA​ACG​GAT​TTG	GGG​GTC​GTT​GAT​GGC​AAC​A	95

### Gut microbiota analysis

Colon fecal microbial DNA was extracted using the HiPure Stool DNA Kits (Magen, Guangzhou, China) according to the manufacturer’s protocols. The final DNA concentration and purification were determined by a NanoDrop 2000 UV-vis spectrophotometer (Thermo Fisher Scientific, United States), and DNA quality was measured by 1% agarose gel electrophoresis. The V3-V4 regions of the bacterial 16S rRNA were amplified by a PCR system (ETC.,811, Dongsheng Xingye Scientific Instrument Co., LTD., China) using primers 341F (5′-CCTACGGGNGGCWGCAG -3′) and 806R (5′-GGACTACNVGGGTWTCTAAT -3′). The second round of amplifications were purified using AMPure XP Beads, quantified using the ABI StepOnePlus RealTime PCR System (Life Technologies, United States) and sequenced according to the PE250 pattern pooling of the Novaseq 6000. The clean tags were clustered into operational taxonomic units (OTUs) of ≥ 97% similarity using UPARSE (version 9.2.64) pipeline. All chimeric tags were removed using UCHIME algorithm and finally obtained effective tags for further analysis. The tag sequence with highest abundance was selected as representative sequence within each cluster. Chao1, ACE, Shannon and Simpson index were calculated in QIIME (version 1.9.1). Multivariate statistical technique PCoA (principal coordinates analysis) of (Un) weighted unifrac, jaccard and bray-curtis distances were generated in R project Vegan package (version 2.5.3) and plotted in R project ggplot2 package (version 2.2.1). Biomarker features in each group were screened by LefSe software (version 1.0). The KEGG pathway analysis of the OTUs/ASV was inferred using Tax4Fun (version 1.0).

### Statistical analysis

All data are presented as mean ± SEM. Comparisons between two groups were performed using the two independent samples *t*-test (Wilcoxon rank test was performed when the parameter test was not satisfied). Comparisons between multiple groups were performed using One-way ANOVA and Tukey test. *p*-value <0.05 was considered statistically significant.

## Results

### Obesity and depression comorbidity model validation

**FIGURE 2:**
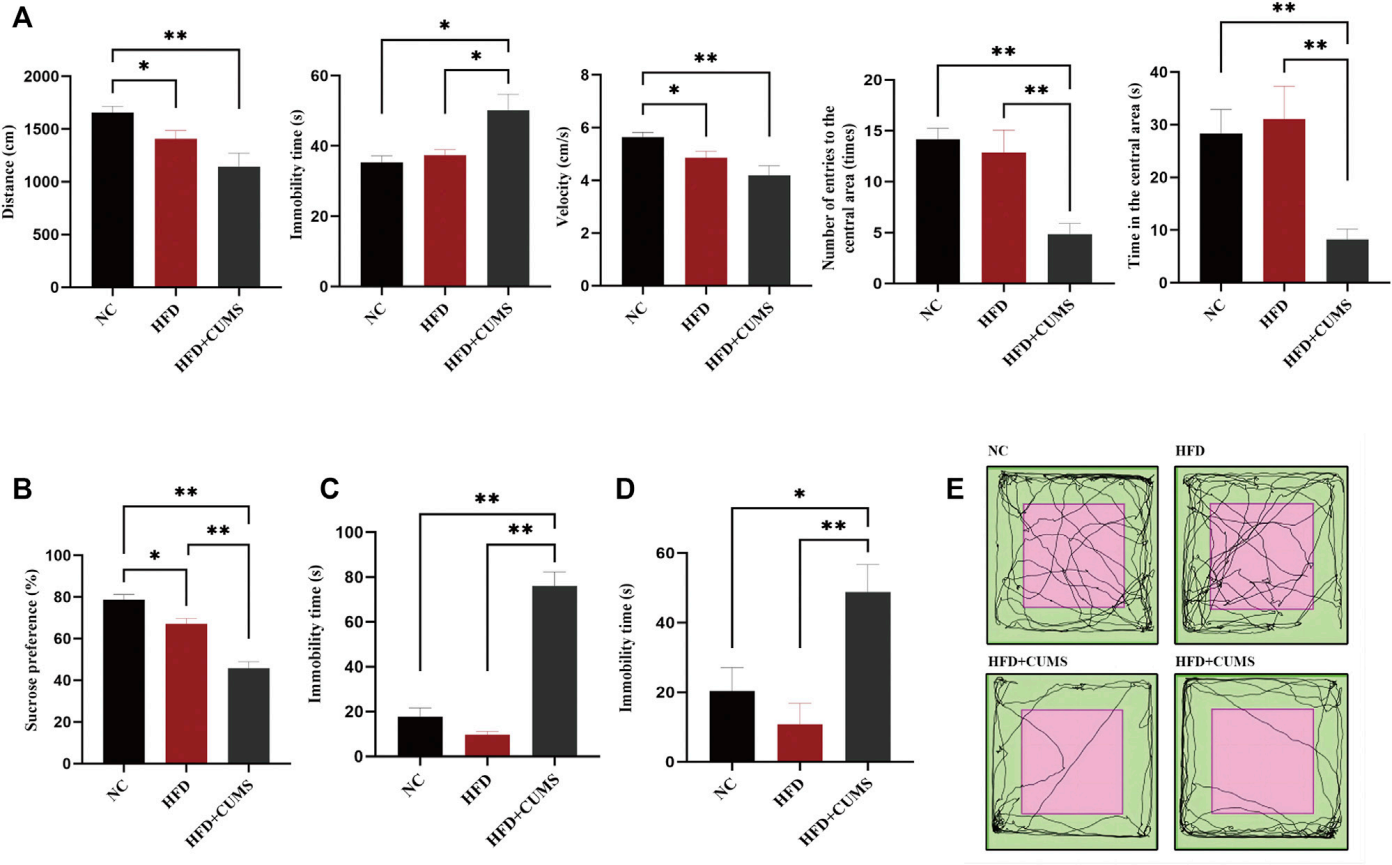
Obesity-Co-Morbid Depression model was established successfully (*n* = 8–9/group). **(A)** Locomotion activity scores in OFT. **(B)** Sucrose preference in SPT. **(C)** Time of immobility in FST. **(D)** Time of immobility in TST. **(E)** Activity tracks in OFT. Data are shown as the mean ± SEM. *p < 0.05, **p < 0.01 (Statistical comparisons between the two groups were performed using the two independent samples *t*-test. Comparisons between multiple groups were performed using One-way ANOVA and Tukey test.).

**FIGURE 3:**
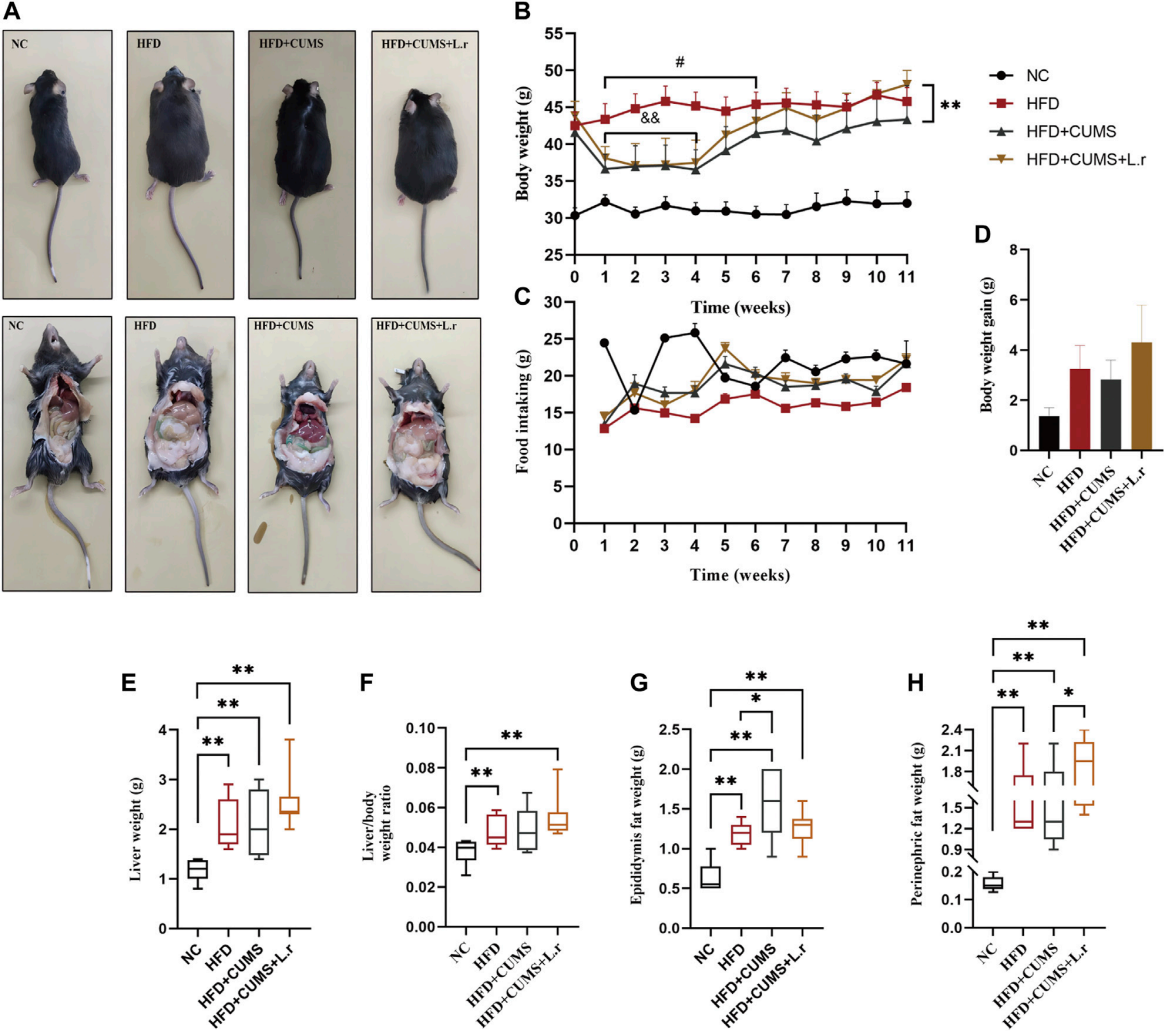
Gavaged with *Lactobacillus reuteri* affected the body weight in comorbid mice (*n* = 8–10/group). **(A)** Body size and fat accumulation of mice. **(B)** Body weight. **(C)** Food intake. **(D)** Body weight gain. **(E)** Liver weight. **(F)** Liver/body weight ratio. **(G)** Epididymis fat weight. **(H)** Perinephric fat weight. Data are shown as the mean ± SEM. and&*p* < 0.01 vs. the HFD group; #*p* < 0.05 vs. the HFD + CUMS group; *p < 0.05, **p < 0.01 (Statistical comparisons between the two groups were performed using the two independent samples *t*-test. Comparisons between multiple groups were performed using One-way ANOVA and Tukey test.).

C57BL/6 mice were fed with normal diet or high-fat diet for 8 weeks. The weight of mice in the HFD group was significantly heavier than that in the NC group (*p* < 0.01; Figure 3B), which suggested that obesity was induced with HFD in mice. After 4 weeks of CUMS, the behavior tests including the OFT, SPT, TST and FST were used to evaluate the influence of the CUMS interventions. As per OFT results, the number and time of entering into the central area in the HFD + CUMS sub-group was decreased as compared to HFD sub-group (*p* < 0.01; Figures 2A,E). The velocity of movement and total distance were also slightly decreased in the HFD + CUMS sub-group when compared to the HFD sub-group, although the differences were not significant (*p* > 0.05;Figure 2A), while the time of immobility was significantly increased in the HFD + CUMS sub-group as compared to HFD sub-group (*p* < 0.05; Figure 2A). Moreover, the sucrose preference was significantly lower in the HFD + CUMS sub-groups in SPT compared to the HFD sub-group, and the immobility time was significantly increased in FST and TST (*p* < 0.01; Figures 2B–D). Noteworthy, the sucrose preference (*p* < 0.05) was decreased in the HFD sub-group compared to the NC group, while immobility time (*p* > 0.05) was not significantly different in the FST and TST (Figures 2B–D). The results showed that CUMS might exacerbate the depressive-like behaviors in obesity mice and the obesity and depression comorbidity model was successfully built.

### Effect of *Lactobacillus reuteri* on body weight, insulin resistance and blood lipids in comorbid mice

Slight weight loss of mice was observed in HFD + CUMS sub-group and HFD + CUMS + L.r sub-group in the period (1–4 weeks) as compared to that of the HFD sub-group (*p* < 0.01; Figures 3A,B). The food intakes in the HFD + CUMS sub-group and HFD + CUMS + L.r sub-group were higher than those in the HFD sub-group (Figure 3C). At week 11, *L. reuteri* gavage increased body weight, body weight gain, liver weight, liver/body weight ratio and perinephric fat weight in HFD + CUMS + L.r sub-group compared with those in HFD sub-group and HFD + CUMS sub-group (Figures 3D–H).

**FIGURE 4:**
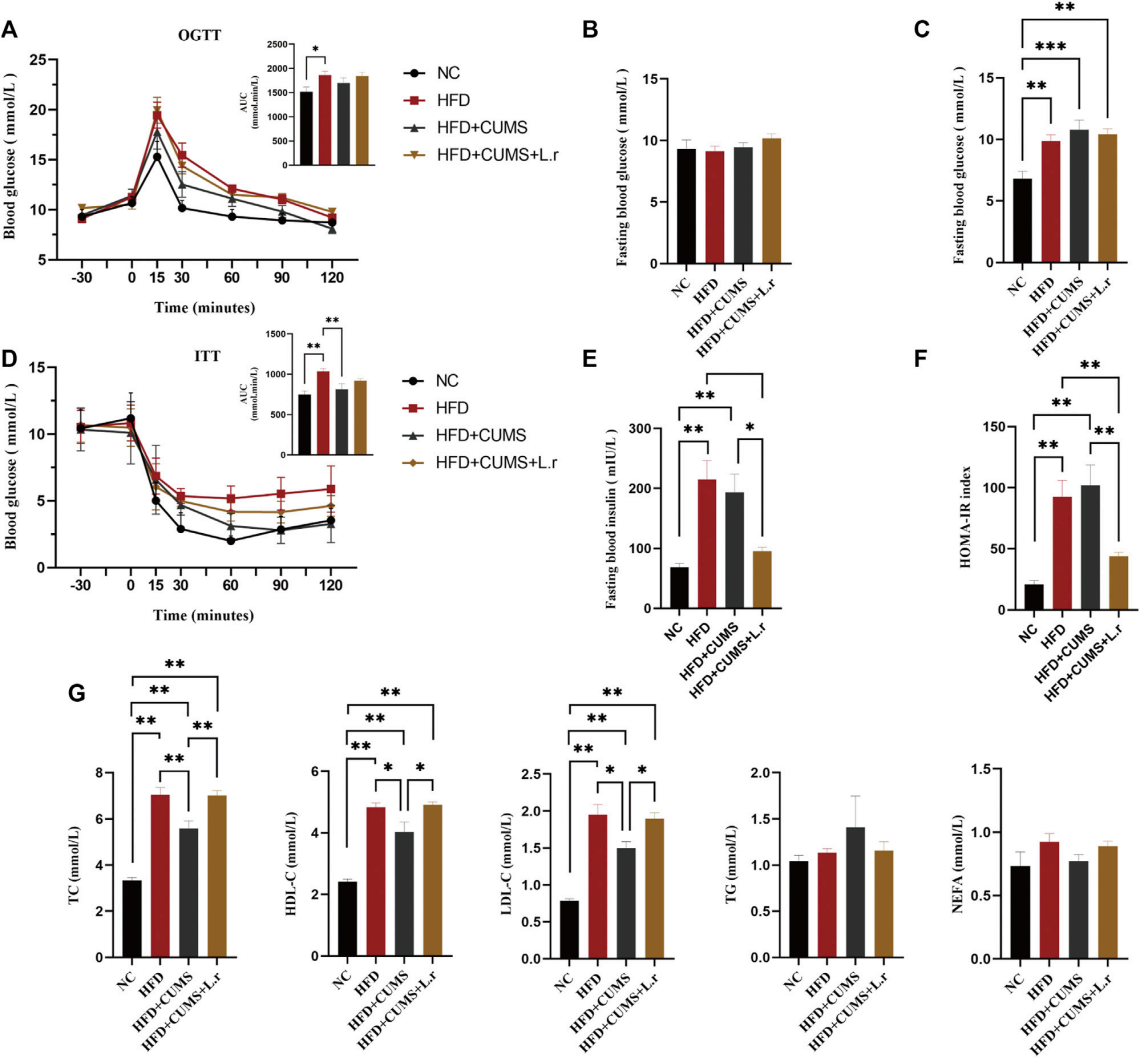
Gavaged with *Lactobacillus reuteri* resisted the development of diabetes and improved the dyslipidemia (*n* = 6–8/group). **(A)** Oral glucose tolerance test. **(B)**Fasting 5-h blood glucose. **(C)** Fasting 12-h blood glucose. **(D)** Insulin tolerance test. **(E)** Fasting blood insulin. **(F)** Homeostasis model assessment (HOMA)-insulin resistance (IR) index. **(G)** Serum Lipid Indicators. Data are shown as the mean ± SEM. *p < 0.05, **p < 0.01 (Statistical comparisons between the two groups were performed using the two independent samples *t*-test. Comparisons between multiple groups were performed using One-way ANOVA and Tukey test.).

The results also demonstrated that the area under the curve (AUC) values in the oral glucose tolerance test (OGTT; Figure 4A) and insulin tolerance test (ITT; Figure 4D) in mice in the HFD group are slightly higher compared to those in the NC group. Although there were no significant differences in fasting 5 h blood glucose in HFD and NC group (Figure 4B), significantly higher fasting 12 h blood glucose in the HFD group were observed as compared to those in the NC group (*p* < 0.01; Figure 4C). Besides, the fasting blood insulin levels and HOMA-IR index were reduced in the HFD + CUMS + L.r sub-group compared to those in HFD + CUMS sub-group, and there were a slightly elevated levels of HOMA-IR index in the HFD + CUMS sub-group compared to the HFD sub-group (*p* < 0.05; Figure 4E; *p* < 0.01; Figure 4F). These data suggested that the CUMS increase the risk of developing diabetes in obese mice, and *L. reuteri* might retard the development of diabetes.

Moreover, *L. reuteri* gavage might improve the dyslipidemia associated with obesity. Compared to the HFD sub-group, HDL-C level was reduced in the HFD + CUMS sub-group while increased in HFD + CUMS + L.r sub-group (*p* < 0.05; Figure 4G). TG level was lower in HFD + CUMS + L.r group compared to that in the HFD + CUMS group in spite of no significant difference (*p* > 0.05; Figure 4G). However, *L. reuteri* gavage did not improve the other blood lipid indexes such as TC, LDL-C and NEFA.

### Effect of *Lactobacillus reuteri* on depressive-like behavior in comorbid mice

**FIGURE 5:**
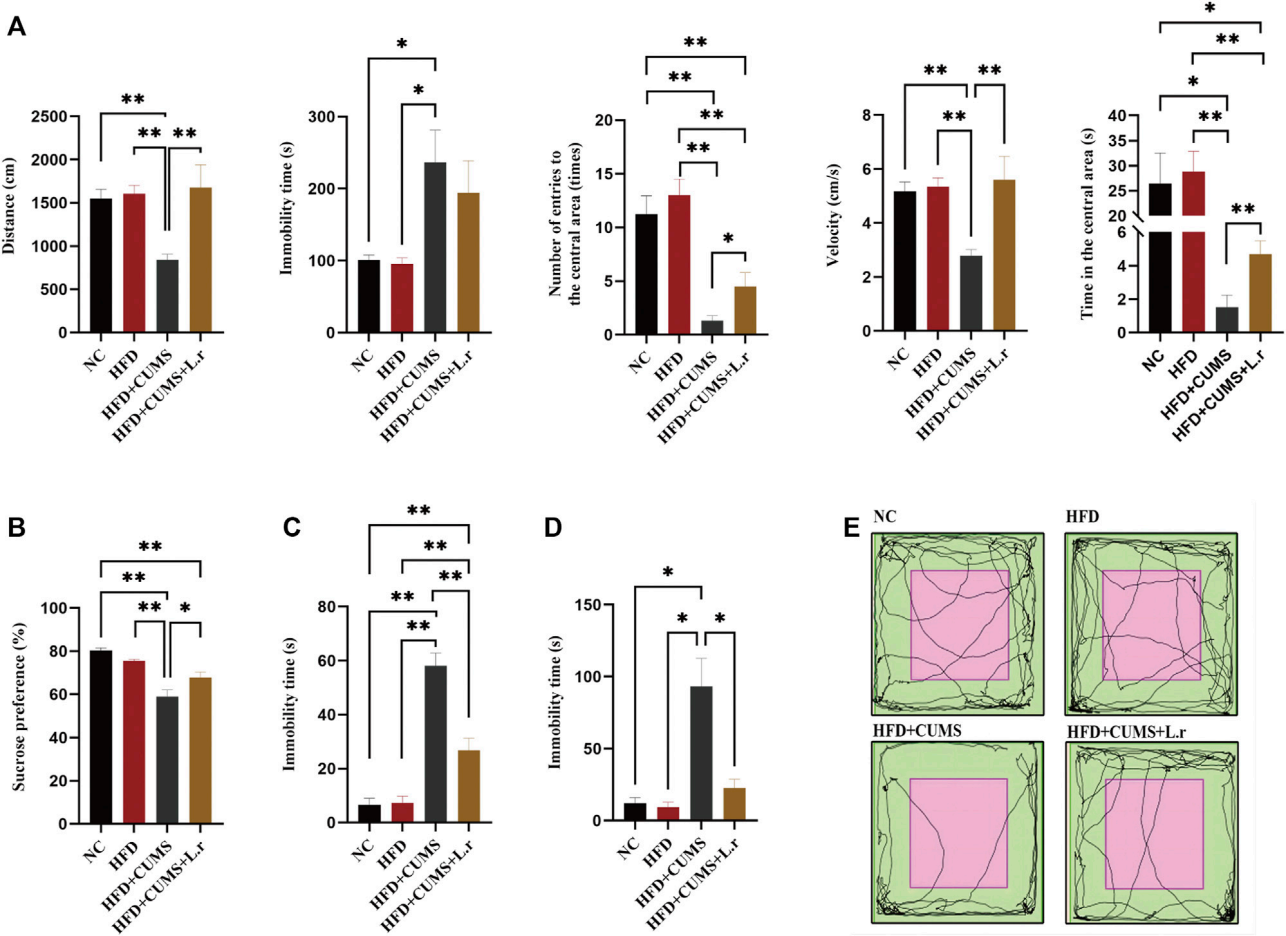
Gavaged with *Lactobacillus reuteri* attenuated depressive-like behavior (*n* = 8–9/group). **(A)** Locomotion activity scores in OFT. **(B)** Sucrose preference in SPT. **(C)** Time of immobility in FST. **(D)** Time of immobility in TST. **(E)** Activity tracks in OFT. Data are shown as the mean ± SEM. *p < 0.05, **p < 0.01 (Statistical comparisons between the two groups were performed using the two independent samples *t*-test. Comparisons between multiple groups were performed using One-way ANOVA and Tukey test.).

The number and time of entering into the central area, velocity, total distance was increased (*p < 0.01 ∼ 0.05*), while the immobility time (*p > 0.05*) was slightly decreased in the HFD + CUMS + L.r sub-group mice as compared to those in the HFD + CUMS sub-group (Figures 5A,E). In addition, the sucrose preference was significantly increased and the immobility time was significantly decreased in the HFD + CUMS + L.r sub-group mice as compared to those in the HFD + CUMS sub-group (*p < 0.01*) (Figures 5B–D). The above results indicated that *Lactobacillus reuteri* might alleviate the depression-like behavior in obesity and depression comorbid mice.

### Effect of *Lactobacillus reuteri* on inflammation in comorbid mice

**FIGURE 6:**
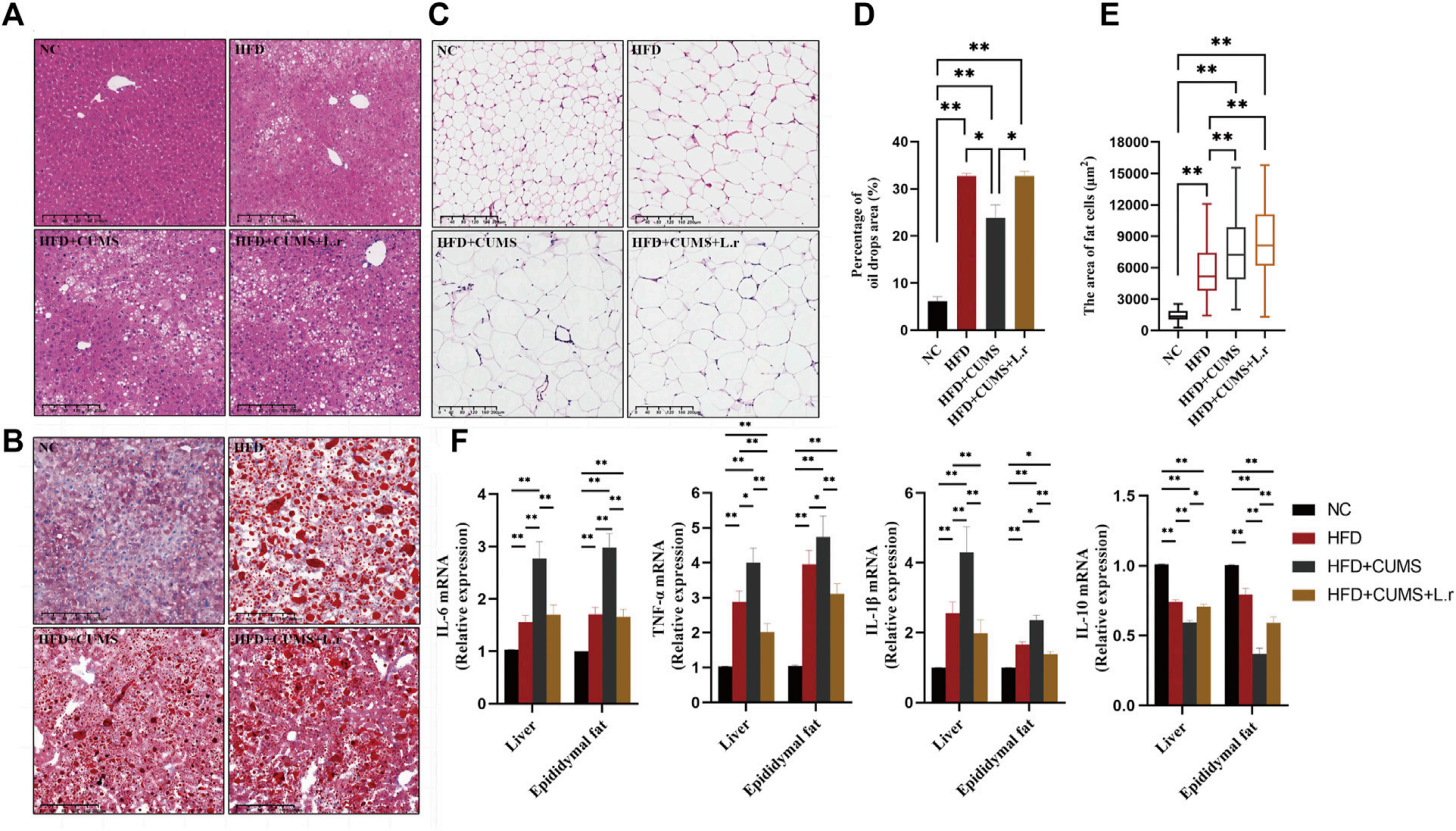
Gavaged with *Lactobacillus reuteri* reduced inflammation in liver and adipose tissues (*n* = 8–9/group). **(A)** H&E staining of the liver tissues. **(B)** Oil Red O staining of the liver tissues. **(C)** H&E staining of the epididymis fat tissues. **(D)** Percentage of oil drops area of the liver tissue. **(E)** Fat cells area of the epididymis fat tissue. **(F)** mRNA expression levels of IL-6, TNF-α, IL-1β, and IL-10 in the liver and epididymis fat tissues. Data are shown as the mean ± SEM. *p < 0.05, **p < 0.01 (Statistical comparisons between the two groups were performed using the two independent samples *t*-test. Comparisons between multiple groups were performed using One-way ANOVA and Tukey test.).

HFD feeding the mice increased the inflammatory response in the liver and adipose tissues. In liver tissue, irregularly arranged liver lobules and cords, hepatocytes of unequal size, hepatocyte degeneration, fatty deposits and marked inflammatory cell infiltration were seen in all sub-groups except the NC group. The pathological changes of liver tissue were improved in HFD + CUMS + L.r sub-group as compared to those in HFD + CUMS sub-group (Figure 6A). Significant oil droplets deposition in the liver tissue were seen in all sub-groups except the NC group (Figures 6B,D). In the adipose tissues, enlarged, irregularly-arranged and unevenly-sized adipocytes were observed in other 3 sub-groups as compared to those in the NC group (Figures 6C,E). *Lactobacillus reuteri* gavage did not contribute positive effect on oil deposition or fat cell distribution. Meanwhile in the later experiment assay, pro-inflammatory cytokines as well as the anti-inflammatory cytokine in the liver and adipose tissues demonstrated various data. The expression levels of pro-inflammatory factors interleukin-6 (IL-6), interleukin-1-beta (IL-1_β_) and tumor necrosis factor-alpha (TNF_α_) were increased in the other 3 sub-groups compared to the NC group. Among them the levels of IL-6, IL-1_β_, and TNF-_α_ were significant higher in the HFD + CUMS group compared to those in HFD + CUMS + L.r sub-group (*p < 0.01*) (Figure 6F). The inverse result was also seen with interleukin-10 (IL-10) (*p < 0.01*) (Figure 6F). Our results showed that CUMS might exacerbate the inflammation of the liver and adipose caused by HFD, while *L. reuteri* gavage might alleviate these alterations through anti-inflammatory effect.

### Effect of *Lactobacillus reuteri* on intestinal tight junctions in comorbid mice

**FIGURE 7:**
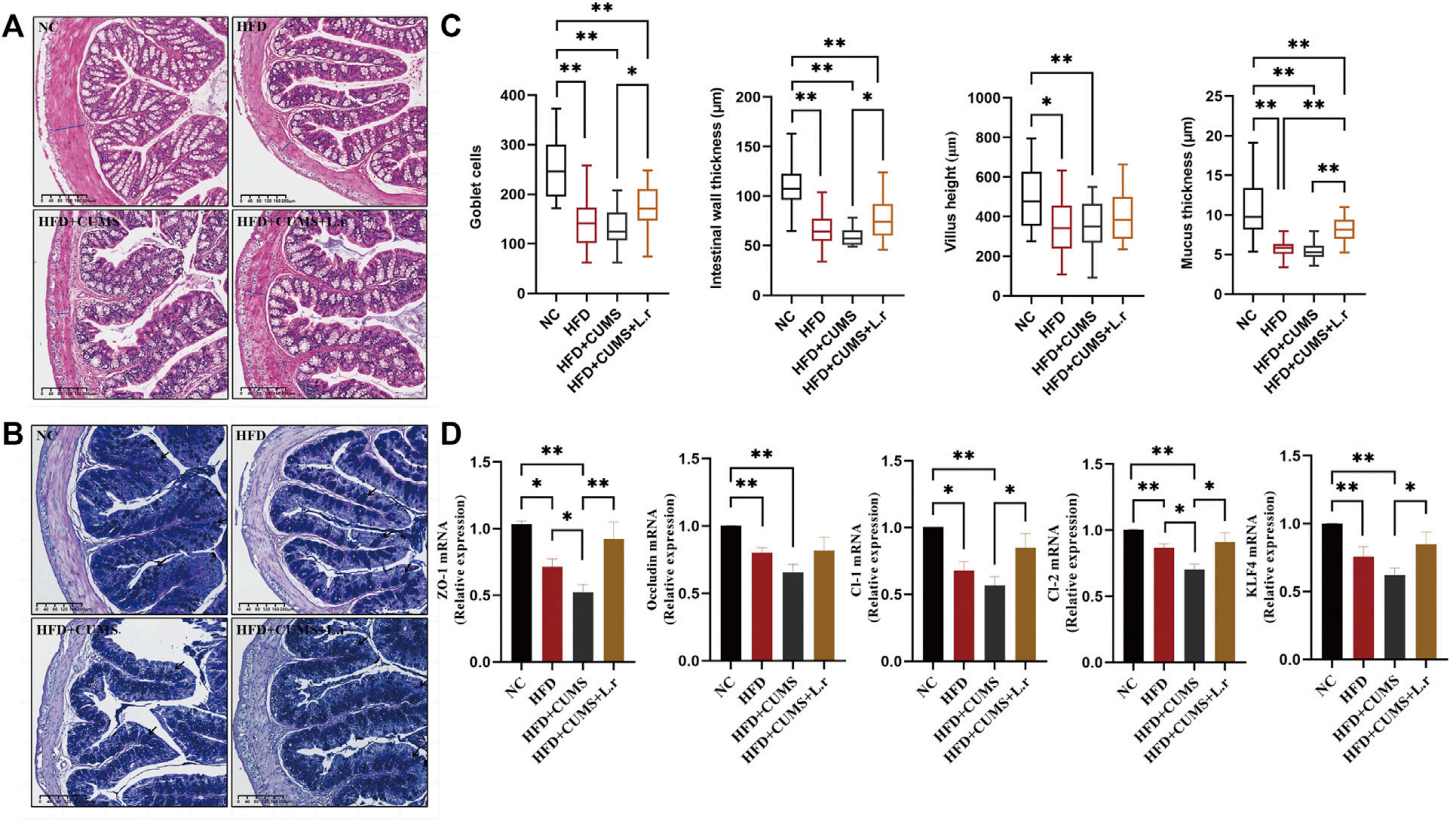
Gavage with *Lactobacillus reuteri* improved intestinal tight junctions (*n* = 8–9/group). **(A)** H&E staining of the colon tissues. **(B)** AB-PAS staining of the colon tissues. **(C)** Number of goblet cells, intestinal wall thickness, villus height and intestinal mucus thickness of the colon tissues. **(D)** mRNA expression levels of ZO-1, Occludin, Claudin-1, Claudin-2, and KLF4 in the colon tissues. Data are shown as the mean ± SEM. *p < 0.05, **p < 0.01 (Statistical comparisons between the two groups were performed using the two independent samples *t*-test. Comparisons between multiple groups were performed using One-way ANOVA and Tukey test.).

H&E and AB-PAS colon staining showed that morphological structure of villus with incomplete goblet cells arrangement were seen in all sub-groups except the NC group. The intestinal wall thickness, villus height, goblet cell quantity and mucus thickness were decreased obviously most in the CUMS + HFD sub-group. These changes were reversed significantly by *L. reuteri* in the HFD + CUMS + L.r sub-group (*p < 0.05*) (Figures 7A–C).

The mRNA expression of tight-junction proteins such as ZO-1, Occludin, Claudin-1 and Claudin-2 in the colon (Figure 7D) showed that the expression levels of ZO-1, Occludin, Claudin-1, and Claudin-2 were decreased in HFD group compared to the NC group (*p < 0.01*). Among the 3 sub-groups, the lowest expressions were observed in the HFD + CUMS sub-group, while a significant increase was seen in the HFD + CUMS + L.r sub-group in comparison to the HFD + CUMS sub-group (*p < 0.05*). Besides, the expression of KLF4 was also reduced in the HFD and the HFD + CUMS sub-groups and upregulated in HFD + CUMS + L.r sub-group (*p < 0.05*). The above results indicated that CUMS might aggravate the disruption of intestinal tight junctions caused by HFD. *Lactobacillus reuteri* gavage might alleviate such alterations and have an overall protective effect on the intestine.

### Effect of *Lactobacillus reuteri* on microbiome dysbiosis in comorbid mice

**FIGURE 8:**
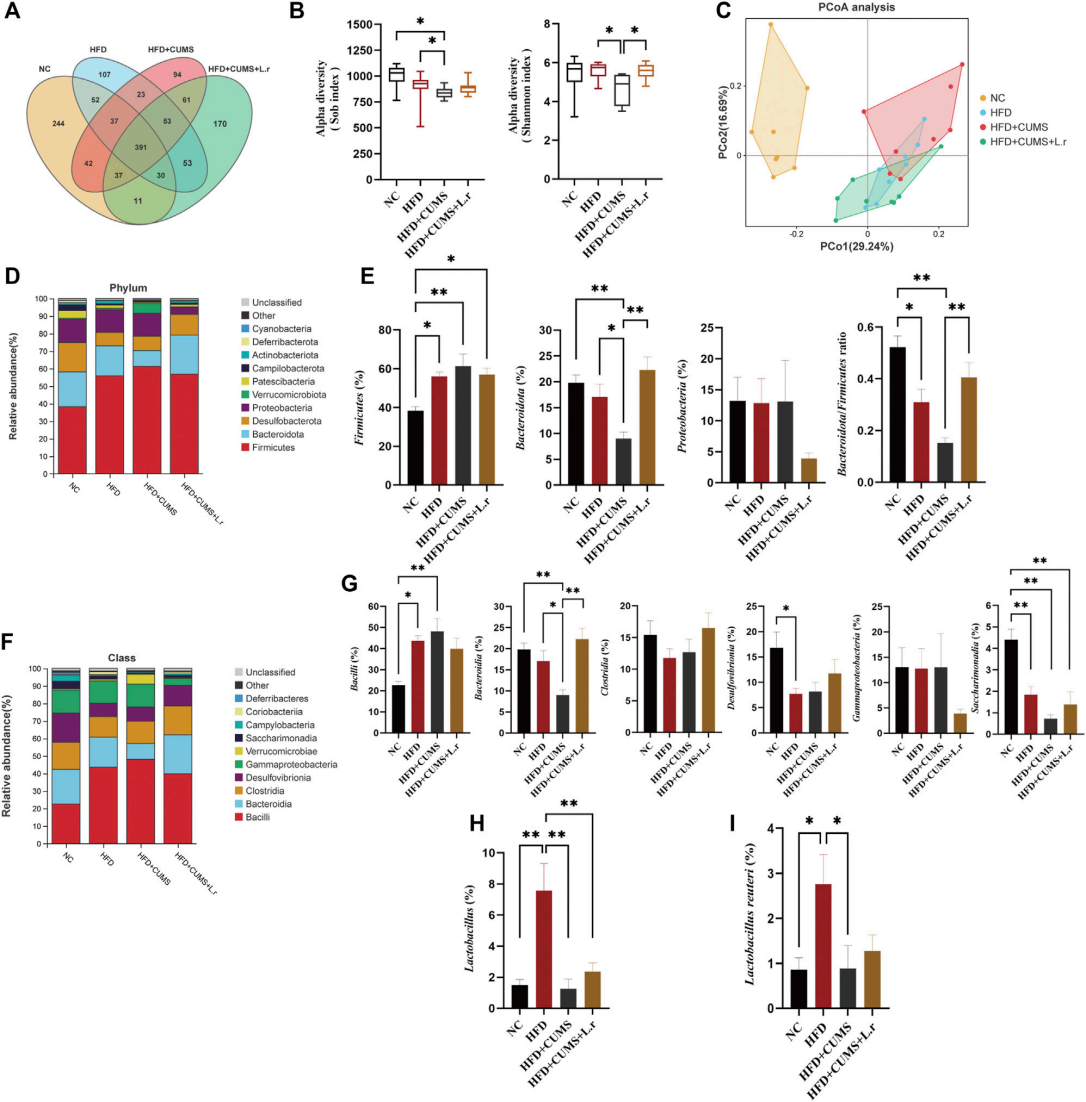
Gavage with *Lactobacillus reuteri* reshaped the microbiome dysbiosis (*n* = 8/group). **(A)** OTU Venn diagram between 4 groups. **(B)** Sob and Shannon indexes of the gut microbiota. **(C)** Weighted UniFrac PCoA analysis of the gut microbiota. **(D)** Phylum-level distribution of fecal microbiota. **(E)** Relative abundance of the phyla *Firmicutes*, *Bacteroidetes*, *Proteobacteria* and *Bacteroidetes*/*Firmicutes* ratio. **(F)** Class-level distribution of fecal microbiota. **(G)** Relative abundance of identified differential abundant bacterial groups at class level. **(H)** Relative abundance of *Lactobacillus* at genus level. **(I)** Relative abundance of *Lactobacillus reuteri* at species level. Data are shown as the mean ± SEM. *p < 0.05, **p < 0.01 (Statistical comparisons between the two groups were performed using the two independent samples *t*-test. Comparisons between multiple groups were performed using One-way ANOVA and Tukey test.).

To explore the possible protective mechanism of *Lactobacillus reuteri* strain 8008 on comorbid mice, 16S rRNA sequencing analysis was conducted to assess alterations in the gut microbiota. First, Venn diagram was used to evaluate the similarity and consistency of the over lapping operational taxonomic units (OTUs) of samples. 391 OTUs were shared in all groups, and 244, 107, 94, 170 were respectively unique to the NC group and HFD, HFD + CUMS and HFD + CUMS + L.r sub-groups. The exclusive OTUs in the HFD + CUMS sub-group were decreased compared to the HFD sub-group. And the exclusive OTUs in the HFD + CUMS + L.r sub-group were increased compared to the HFD + CUMS sub-group (Figure 8A). Then the α-diversity of the colonic fecal microbiome was analyzed. The Sob and Shannon indices were selected to assess community richness and diversity, and results showed that HFD reduced the richness and diversity of the community. CUMS exacerbated the reduction in community richness and diversity in HFD + CUMS sub-group compared to that in the HFD group, while the *L. reuteri* gavage reversed these reductions in HFD + CUMS + L.r sub-group (Figure 8B). Then the β-diversity was studied with a Weighted Unifrac PCoA analysis (Pco1 29.24%, Pco2 16.69%) to describe the difference of gut microbial communities in structural alterations and species complexity. The apparent differences existed in the gut microbiota structure among 4 groups (Figure 8C). The gut microbiota structure in HFD + CUMS + L.r sub-group was more similar to that in HFD sub-group as compared to that in HFD + CUMS sub-group. The results showed that *L. reuteri* might reverse gut microbiota structural changes in obesity and depression comorbid mice.

To further understand the specific changes in the microbiota, top 10 species with the highest abundance of each sample were selected at phylum and class levels based on the species annotation results of OTUs. At the phylum level, *Firmicutes* and *Bacteroidetes* were the dominant phyla amongst all groups, accounting for over 60% of the total sequences (Figure 8D). Besides, the relative abundance of the *Firmicutes* were increased in other 3 sub-groups as compared to those in the NC group, obviously most in the CUMS + HFD sub-group, and such change was slightly reversed by *L. reuteri* gavage as compared to the HFD + CUMS sub-group. In contrast, the phylum *Bacteroidetes* showed an opposite trend. Moreover, the *Bacteroidetes*/*Firmicutes* ratio was lowest in the HFD + CUMS sub-group compared to that in HFD and in HFD + CUMS + L.r sub-groups (Figure 8E). In addition, the relative abundance of *Proteobacteria* was lower in the HFD + CUMS + L.r sub-group compared with the other three sub-groups. At the class level, ten classes including *Bacilli*, *Bacteroidia*, *Clostridia*, *Desulfovibrionia*, *Gammaproteobacteria* and *Saccharimonadia* were found in all samples (Figure 8F). Compared to the NC group, the abundance of *Bacteroidia*, *Clostridia*, *Desulfovibrionia* and *Saccharimonadia* was decreased in the HFD and HFD + CUMS sub-groups. In contrast, the abundance of *Bacilli*, which belongs to *Firmicutes*, showed an opposite trend. What’s more, the abundance of *Gammaproteobacteria* was dropped in the HFD + CUMS + L.r sub-group compared with the other three groups (Figure 8G). The relative abundance of *Lactobacillus* (genus level) and *Lactobacillus reuteri* (species level) were decreased in the HFD + CUMS sub-group compared to the HFD group, and slightly increased in the HFD + CUMS + L.r sub-group compared to the HFD + CUMS group. (Figures 8H,I). The above results showed that CUMS might aggravate the microbiome dysbiosis caused by HFD, and *L. reuteri* gavage might improve such microbiome dysbiosis.

### Biomarker analysis and Tax4Fun microbiota’s functional predictions

**FIGURE 9:**
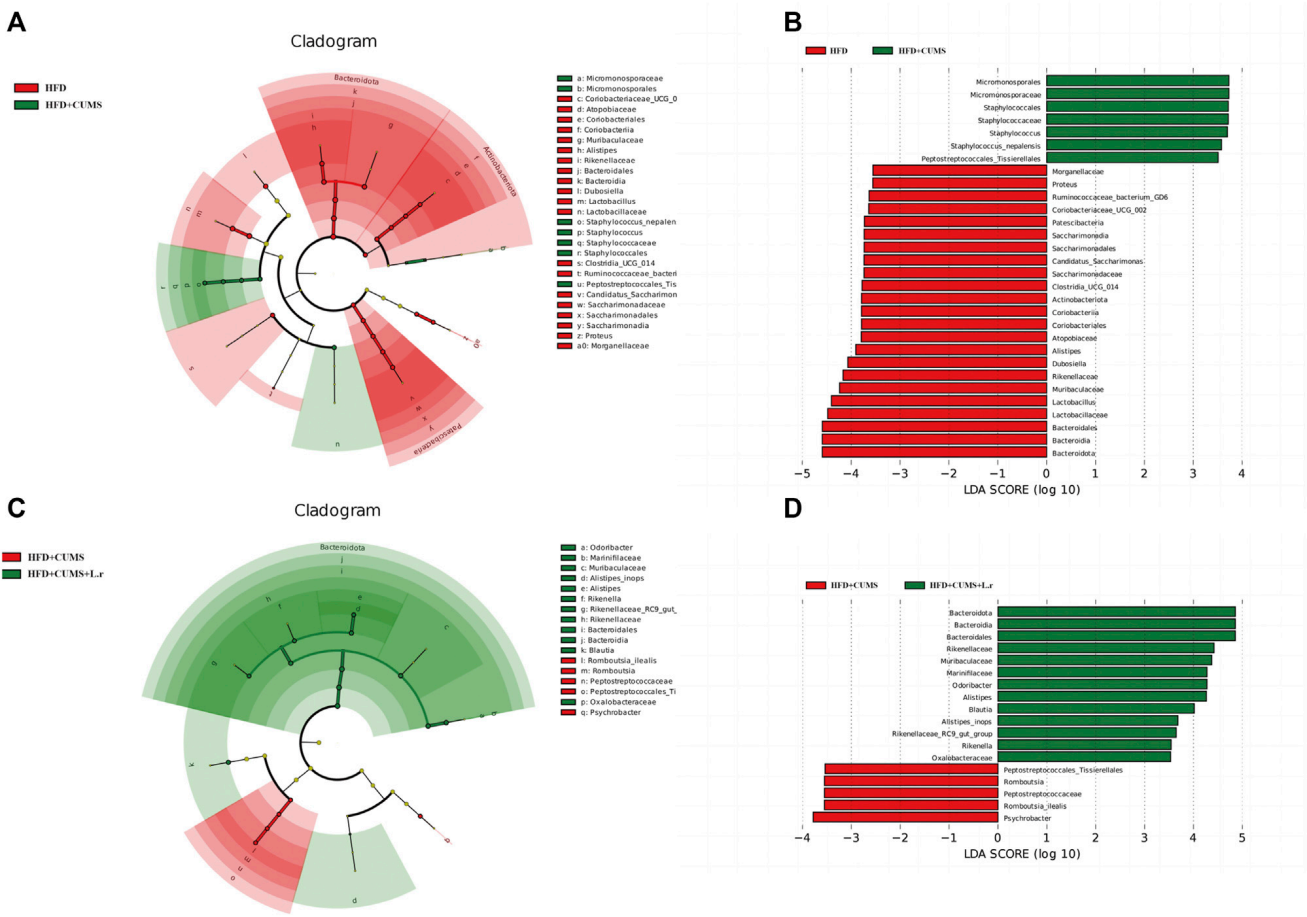
Biomarker analysis of the gut microbiota community (*n* = 8/group). **(A)** LEfSe cladogram represents the taxa enriched in the HFD group (Red) and HFD + CUMS group (Green). **(B)** Discriminative biomarkers with an LDA score >3.5 between the HFD group (Red) and HFD + CUMS group (Green). **(C)** LEfSe cladogram represents taxa enriched in the HFD + CUMS group (Red) and HFD + CUMS + L.r group (Green). **(D)** Discriminative biomarkers with an LDA score >3.5 between the HFD + CUMS group (Red) and HFD + CUMS + L.r group (Green).

Biomarkers can be used as potential indicator species in disease detection and they might be potentially used for personalized therapy. LEfSe analysis was performed at the species level to explore the prognostic biomarkers from the microbial abundance profiling. The result showed that the abundance of *Micromonosporales*, *Staphylococcales* and *Staphylococcus* [LDA score (−log10) > 3.5] was increased, and decreased the abundance of *Bacteroidota*, Lactobacillaceae, Muribaculaceae, Rikenellaceae, *Alistipes* and Atopobiaceae was decreased in HFD + CUMS sub-group compared to the HFD sub-group [LDA score (−log10) > 3.5] (Figures 9A,B). As for HFD + CUMS + L.r group, the abundance of *Bacteroidota*, Rikenellaceae, Muribaculaceae, Marinifilaceae and *Blautia* were increased compared to the HFD + CUMS sub-group [LDA score (−log10) > 3.5] (Figures 9C,D).

**FIGURE 10:**
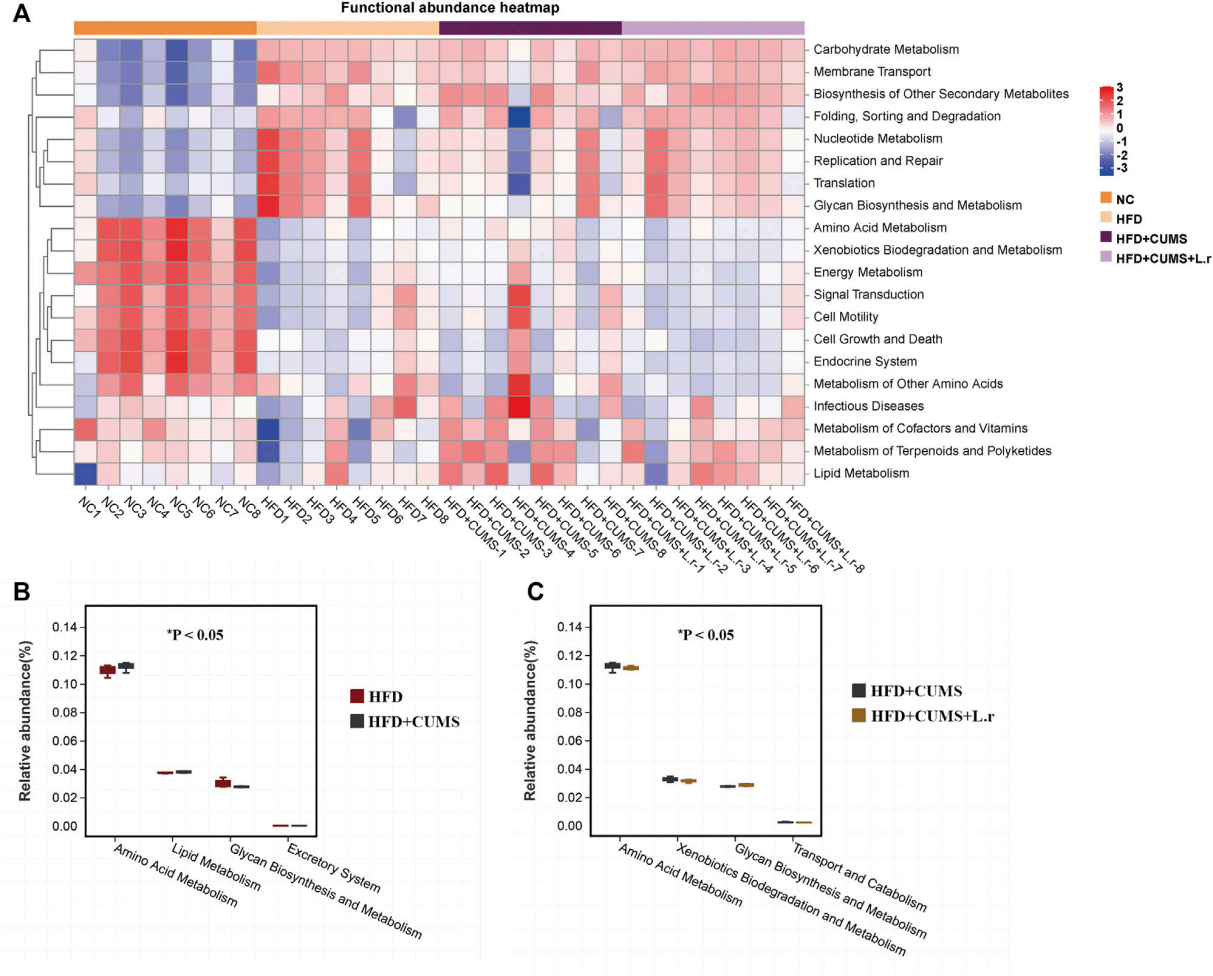
Tax4Fun bacterial community’s functional predictions (*n* = 8/group). **(A)** Functional annotation clustering heat map using Tax4Fun on level 2. **(B)** Functional annotation relative abundance between the HFD and HFD + CUMS groups on level 2. **(C)** Functional annotation relative abundance between the HFD + CUMS and HFD + CUMS + L.r groups on level 2. (Statistical comparisons between the two groups were performed using the Wilcoxon rank test.)

To further explore the functional information associated with changes in the intestinal flora, the clustering heat map was annotated on level 2 by using the Tax4Fun function. According to the KEGG (Kyoto Encyclopedia of Genes and Genomes) Pathway database, the top 20 functional information in the maximum abundance of each group were selected to draw a heat map (Figure 10A). At level 2, the “Amino Acid Metabolism” and “Lipid Metabolism” pathways were increased while the “Excretory System” and “Glycan Biosynthesis and Metabolism” pathways were decreased in the HFD + CUMS sub-group as compared to the HFD sub-group (Figure 10B). The “Amino Acid Metabolism”, “Xenobiotics Biodegradation and Metabolism” and “Transport and Catabolism” pathways were decreased and the “Glycan Biosynthesis and Metabolism” pathway was increased in the HFD + CUMS + L.r sub-group as compared to that in the HFD + CUMS sub-group (Figure 10C).

### Correlations between gut microbiota and tight-junction proteins/bacterial community’s functions

**FIGURE 11:**
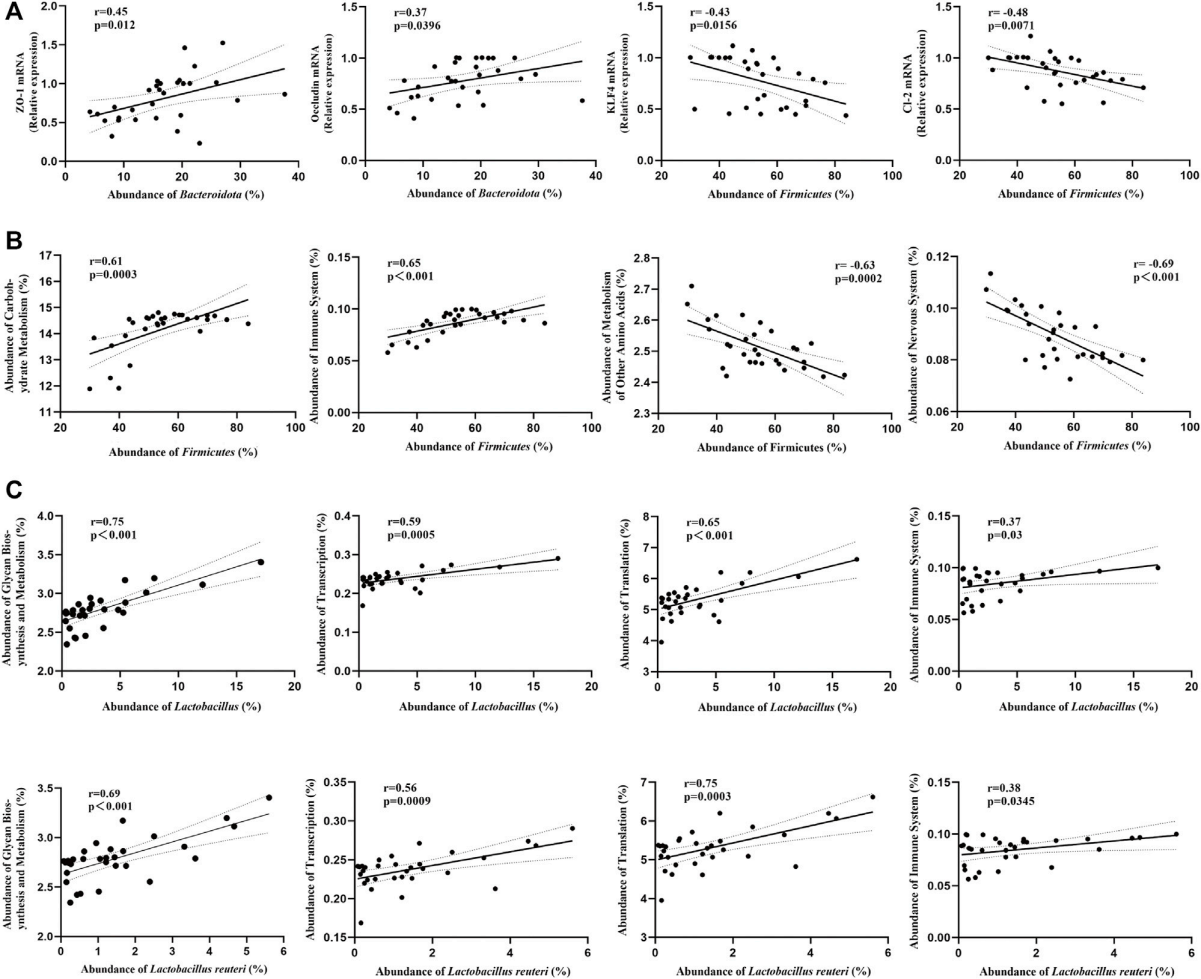
Correlations between gut microbiota and tight-junction proteins/bacterial community’s functions (Pearson’s r correlation and corresponding *p*-value) (*n* = 8/group). **(A)** Correlations between gut microbiota (phylum level) and tight-junction proteins mRNA. **(B)** Correlations between gut microbiota (phylum level) and bacterial community’s functions. **(C)** Correlations between gut microbiota (genus/species level) and bacterial community’s functions.

In order to better understand the relationship between gut microbiota and tight-junction proteins/bacterial community’s functions, we performed the correlation analysis. At phylum level, we found that the mRNA expression of ZO-1 and Occludin were positively correlated with the abundance of *Bacteroidota*, and the mRNA expression of KLF4 and Claudin-2 were negatively correlated with the abundance of *Firmicutes* (Figure 11A). Besides, we found that the “Carbohydrate Metabolism” and the “Immune System” pathways were positively correlated with the abundance of *Firmicutes*, while the “Metabolism of Other Amino Acids” and the “Nervous System” pathways were negatively correlated (Figure 11B). Moreover, at genus/species level, the “Carbohydrate Metabolism”, “Translation”, “Immune System” and the “Transcription” pathways were positively correlated with the abundance of *Lactobacillus* or *Lactobacillus reuteri* (Figure 11C).

## Discussion

Depression is one of the most common comorbidities of chronic diseases such as obesity, and there are a large proportion of the reviews show an association between obesity and depression (Atlantis and Baker, 2008; de Wit et al., 2010; Roberts and Duong, 2013; Jokela et al., 2014). A Meta-analysis indicated that there was a positive link between obesity and depression among adult populations, which appeared to be more marked among women (Abou Abbas et al., 2015). Besides, the occurrence of depression comorbidity in obese patients is heavily influenced by some psychosocial factors. Obese patients tend to suffer from problems with body image, social isolation and self-esteem. Risk factors such as long-term sedentary lifestyles, reduction in time for exercise, increase in high-calorie foods intake, as well as reduction in sleep duration have been the main drivers of obesity. These factors are common in patients with depression, and they are more likely to show bad behaviors including excessive drinking, poor appetite, malnutrition, insomnia and sedentariness (van Gool et al., 2003; van Gool et al., 2007; Milaneschi et al., 2019). The biological mechanisms of genetics, HPA axis, immuno-inflammatory activation and gut microbiota underlying obesity and depression linking have been widely explored (Milaneschi et al., 2019). Long-term HPA axis hyperactivation leads to neuronal damage, and such over-activation can also be found in nearly half of adult obese persons (“hypercortisolistic obesity”) (McEwen, 2007; Wester et al., 2014). Besides, depression and obesity share several dysregulated biological pathways, such as heightened oxidative and disturbed inflammatory pathways (Lopresti and Drummond, 2013). Blood and urine metabolites also differ in depressed and obese patients compared to normal subjects (Xu et al., 2012; Bhattacharyya et al., 2019; Mayengbam et al., 2019; Uemura et al., 2019; Huang et al., 2021). In summary, there exists a strong association between obesity and depression.

In the present study, we first successfully built the obesity and depression comorbidity mice model and investigated the various disease indicators of obesity symptoms under the depression disease comorbidities, as well as exploring the ameliorative or therapeutic effects of *Lactobacillus reuteri* strain 8008 in comorbid mice. The CUMS procedure utilized in our study is widely used in the study of depression for its representation of the pathophysiology of human depression (Hill et al., 2012; Monteggia et al., 2014). Mice were exposed to a series of minor-intensity stressors at unpredictable times over a period of several weeks, the stressors mainly include tilted cage, body restriction, damp bedding, empty cage and other similar unpredictable stresses. The aim of CUMS interventions is the long-term development of depressive states in response to unpredictable stressful stimuli, and these resulted in behavioral changes in mice, including anhedonia (loss of pleasure) and apathy (Wang et al., 2017; Nollet, 2021). Besides, stress increases proinflammatory cytokines in the peripheral and central nervous system (CNS), as well as changes in certain endocrine and neurological variables (Leng et al., 2018; Li et al., 2021; Wang et al., 2021). In our study, after the CUMS interventions, reduced locomotor activity in the OFT, reduced sucrose preference in the SPT as well as increased immobility time in the FST and TST were observed in the HFD + CUMS sub-group when compared to the HFD sub-group. However, it is worth noting from recent studies that high-fat diet (HFD) could induce depressive-like behavior in mice (Yu et al., 2021; Yang et al., 2022), and we did found that the sucrose preference also decreased in the HFD sub-group compared to the NC group, but no corresponding results were presented in the FST and TST. The mice in their studies were fed a HFD for a different duration and age than in our study, we hypothesized that the severity of depressive-like behavior induced by a HFD in mice might be related to these factors. In our study, the CUMS interventions exacerbate the depressive-like behaviors in obesity mice. And the depressive-like behavior of comorbid mice showed improvement after gavage with *Lactobacillus reuteri*, which was in line with some studies (Jang et al., 2019; Xie et al., 2020). Our results indicated that *Lactobacillus reuteri* strain 8008 could contribute to alleviating the aggravation of CUMS-induced depressive-like behavior in obesity and depression comorbidity mice.

Besides, we found an increase in 12 h fasting blood glucose, fasting blood insulin, HOMA index and TG levels and a decrease in HLD-C levels in the HFD + CUMS sub-group compared to the HFD group, and treatment with *Lactobacillus reuteri* strain 8008 improved these alterations. The study had also shown that intake of *Lactobacillus reuteri* might improve incretin and insulin secretion in glucose-tolerant humans (Simon et al., 2015). However, our result showed a potential improvement in ITT and a decrease in the percent of liver oil drops area in HFD + CUMS, as compared to HFD mice. As we all know, the “gold standard” for accurately determining insulin resistance is the hyperinsulinemic-euglycemic clamp (Kim, 2009), however, this technique cannot be routinely implemented in most laboratories. ITT is a simple way to evaluate insulin action *in vivo* in mice, however, it also has limitations. We considered that the result of ITT might be influenced by the hormone-mediated counterregulatory mechanisms to restore blood glucose homeostasis. A combination of data such as fasting blood glucose, fasting insulin, HOMA-IR and HOMA-IS index might be required. Synthesized all data analysis, our results showed that CUMS did exacerbate the insulin resistance in HFD + CUMS group compared to the HFD group, therefore, we considered that the improving ITT in HFD + CUMS group might be influenced by the negative feedback regulation mechanisms, and some studies also showed this same phenomenon (Zhou et al., 2008; Zhang et al., 2020). And according to our result of serum lipid levels, we considered that the reducing percent of liver oil drops area in HFD + CUMS as compared to HFD mice might be influenced by some serum lipid levels such as TC and LDL-C and some metabolic enzymes associated with lipid accumulation. In addition, we noticed that HFD + CUMS group showed lower TC and LDL-C levels, however, the HFD + CUMS group also showed a lower HDL-C level and a higher TG level when compared to the HFD group. It is generally accepted that HFD feeding disturbs serum lipid profile with lower HDL-C level and higher TC, TG and LDL-C levels (Li et al., 2020a). However, the lipid profile of obese mice was not unanimously reflected in the literature. Some studies showed a higher level of HDL-C and a lower level of TG in obese mice when compared to the control mice (Ivanovic et al., 2015). Moreover, some studies hypothesized that the total concentration of lipoprotein increased to transport lipids in serum when fed with HFD (Li et al., 2020b). Inconsistent serum lipid levels were also present in our study, and we considered that the lower levels of TC and LDL-C might influence by some metabolic enzymes associated with lipid accumulation, and the CUMS exposure might play a role in these mechanisms. However, further researches are needed to verify these. Over all, our results suggested that gavage with *Lactobacillus reuteri* might improve glycolipid metabolism, and CUMS-induced depression might have aggravated the glucose and lipid metabolisms in obese mice, as well as increased the risk of developing diabetes.

Studies have shown increased inflammatory symptoms in depressed mice/rats built by CUMS interventions (Song et al., 2018; Yang et al., 2021), and we hypothesized that weight loss in comorbid mice might be associated with increased inflammatory symptoms. To further test our hypothesis, we accessed the gene expression of inflammatory factors in the liver and adipose tissues of mice. The results showed that the levels of IL-6, IL-1β and TNF-α were significantly higher in the HFD + CUMS sub-group compared to those in HFD + CUMS + L.r sub-group. Noteworthy, due to some studies showing that both subcutaneous and epididymal white adipose tissue indicated consistent inflammatory response and autophagy results (Maharjan et al., 2021; Sakane et al., 2021), we only selected the epididymal and perirenal white adipose tissue for testing. However, subcutaneous/inguinal white adipose tissue could more distinctly show differences related to fat lipolysis, and the lack of relevant tissue for testing was a limitation of our study. In a word, the CUMS exposure did exacerbate the inflammatory symptoms in obese mice, while the *Lactobacillus reuteri* intervention alleviated that phenomenon. Additionally, we observed that at week 11, the comorbid mice in the HFD + CUMS + L.r sub-group had higher food intake and body weight compared to the HFD + CUMS sub-group. Although a significantly higher abundance of *Lactobacillus reuteri* in the intestinal flora of obese patients than in normal subjects has been observed in the clinic, we thought that it was not the *Lactobacillus reuteri* that directly caused the body weight gain in mice, but rather the improvement of symptoms such as inflammation and depressive-like symptoms after *Lactobacillus reuteri* gavage, as well as the increase in appetite with a preference for energy-dense food and the normal activities.

The gut microbiota has emerged as a key entry point for research into the pathogenesis and therapeutic treatment of depression and obesity. As the habitat of the gut bacteria, the intestinal barrier plays an important role in various physiological functions such as nutrient metabolism and immune regulation in the human body (Camilleri et al., 2012). Moreover, the integrity of the intestinal epithelium is considered to be the first line of gastrointestinal defense. HFD and CUMS-induced depression have been reported to impair the functions of the intestinal barrier (Cani et al., 2008; Li X. et al., 2019), separately, however, the impact of both comorbidities remains unclear. Thus, we measured the gene expression of the intestinal tight junction proteins of the comorbid mice. Our results showed that HFD did impair the intestinal barrier in mice with reduced mRNA expression of tight junction proteins such as ZO-1, Occludin, Claudin-1 and Claudin-2 in all sub-groups compared to the NC group. Besides, the expression of KLF4, a transcription factor involved in goblet cell differentiation, was also reduced in all sub-groups. And we also found the lowest tight junction proteins expression and the most severe impairment of intestinal barrier functions in the HFD + CUMS sub-group, whereas the levels of ZO-1, Occludin, Claudin-1, Claudin-2 and KLF4 were upregulated in HFD + CUMS + L.r sub-group. Our results indicated that the CUMS exposure exacerbated aggravated the impairment of intestinal barrier functions induced by HFD, and gavage with *Lactobacillus reuteri* might alleviate such impairments. Structural changes in the gut microbiota have been reported in depressed and obese patients (Gomes et al., 2018; Simpson et al., 2021). Recent studies showed that *Lactobacillus reuteri* is able to attach to mucins and intestinal epithelial cells, secrete “Reuterin” which has an antibacterial effect, regulates the distribution of gut microbiota and strengthens the intestinal barrier functions. We hypothesized that CUMS-induced depression altered the abundance of gut bacteria in obese mice, thus aggravating the impairment of the intestinal barrier, while gavage of *Lactobacillus reuteri* resulted in the regulation of the altered abundance of gut bacteria in mice, which in turn alleviated the impairment of barrier functions.

In order to verify our hypothesis, we performed 16S rRNA sequencing analysis of the colon contents to investigate changes in the abundance of colonic gut bacteria. According to the correlation analysis between gut microbiota and tight-junction proteins, we found that the mRNA expression of ZO-1 and Occludin were positively correlated with the abundance of *Bacteroidota*, and the mRNA expression of KLF4 and Claudin-2 were negatively correlated with the abundance of *Firmicutes*. These indicated that the gut bacteria did play an important role in the intestinal barrier functions. Structural changes in the gut microbiota have been reported in depressed and obese patients (Gomes et al., 2018; Simpson et al., 2021). Our α-diversity results showed both intestinal flora richness and evenness were reduced in all sub-groups compared to the NC group, with the lowest in the HFD + CUMS sub-group, whereas increased in the HFD + CUMS + L.r sub-group. Furthermore, the β-diversity results also showed significant differences in gut bacteria structure in all groups, and there was a significant difference between HFD + CUMS and HFD sub-groups, while this difference was attenuated after gavage with *Lactobacillus reuteri*. Studies showed that HFD caused microbiome dysbiosis both in humans and animals, and such disturbance of the “healthy” gut microbiota might drive the development of various chronic diseases, such as obesity and diabetes (Ding et al., 2020). HFD feeding significantly increased the relative abundance of the *Firmicutes* and decreased the relative abundance of the *Bacteroidetes*, indicating that these major phyla might play a role in obesity-related disease indicators (Zhang et al., 2020). In line with this study, we found a higher abundance of *Firmicutes* among all sub-group compared to the NC group. In some studies, obesity was characterized by an impaired *Bacteroidetes*/*Firmicutes* ratio, and depressed patients showed a higher abundance of *Firmicutes* and lower microbiota phylogenetic diversity as compared to normal subjects (Turnbaugh et al., 2006; Zheng et al., 2016). Moreover, *Bacteroidetes*/*Firmicutes* ratio was an indicator associated with inflammation reported in some studies (Gu et al., 2020). In our study, we found that at the Phylum level, the ratio of *Bacteroidetes*/*Firmicutes* was lower in all sub-group than in the NC groups, with the lowest in the HFD + CUMS sub-group, while significantly increased in the HFD + CUMS + L.r sub-group. Our results suggested that the *Bacteroidetes*/*Firmicutes* ratio might correlate with improvements in inflammation, depression-like behavior and intestinal permeability in obesity and depression comorbidity disorders. Besides, we also observed that the abundance of *Proteobacteria* was lowest in the HFD + CUMS + L.r sub-group than in the other groups, and we thought that this might be related to the anti-harmful bacterial effect exhibited by the “Reuterin” secreted from *Lactobacillus reuteri*. At the Class level, we likewise observed changes in the abundance of gut bacteria: the abundance of *Bacteroidia*, *Clostridia*, *Desulfovibrionia* and *Saccharimonadia* decreased in the HFD and HFD + CUMS sub-groups compared to the NC group, in contrast, the abundance of these bacteria were increased in the HFD + CUMS + L.r sub-group. And the abundance of *Bacilli* showed an opposite trend. It is worth noting the previous study reported that the increase in *Bacilli* abundance exerts an anti-inflammatory effect, whereas the increase in *Clostridia* abundance causes a pro-inflammatory effect (Song et al., 2021), inconsistent with our results. Our results showed that *Bacilli* abundance was positively associated with increased tissue inflammation, while the opposite was observed for changes in *Clostridia* abundance. Furthermore, at the Genus and Species levels, we observed the highest levels of *Lactobacillus* and *Lactobacillus reuteri* in the HFD sub-group, in line with clinical findings reported that obese patients have higher abundances of *Lactobacillus* and *Lactobacillus reuteri* than the normal subjects (Gérard, 2016). However, the abundances of *Lactobacillus* and *Lactobacillus reuteri* in the gut of obese mice decreased significantly under CUMS-induced depression comorbidity, and after gavage of *Lactobacillus reuteri* strain 8008, the abundances of *Lactobacillus* and *Lactobacillus reuteri* both slightly increased. Our study indicated that *Lactobacillus reuteri* strain 8008was able to regulate changes in the abundances of the microbiota in obesity and depression comorbidity mice.

To understand the functional interactions between gut bacteria and hosts, we investigated the predicted functional differences in KEGG pathways. We observed that several predicted pathways, such as Carbohydrate Metabolism, Membrane Transport, Biosynthesis of Other Secondary Metabolites, Nucleotide Metabolism, Glycan Biosynthesis and Metabolism, Endocrine System, signal transduction and Lipid Metabolism, differed among four groups. Glycan biosynthesis and metabolism is an important component under the metabolism classification, in our study, we observed that the “Glycan Biosynthesis and Metabolism” pathway was decreased in the HFD + CUMS sub-group compared to the HFD sub-group, while increased in the HFD + CUMS + L.r sub-group. Notably, *Lactobacillus reuteri* can produce glucans and fructans during fermentation, and one of these glucans, α-1,4/1,6 glucan, appears to have a beneficial impact on insulin and blood glucose levels in humans (Kralj et al., 2002), and the gut bacteria associated with the “Glycan Biosynthesis and Metabolism” pathway appear to play a part in this process. Besides, we found that the “Carbohydrate Metabolism” and the “Immune System” pathways were positively correlated with the abundance of *Firmicutes*, while the “Metabolism of Other Amino Acids” and the “Nervous System” pathways were negatively correlated. At genus and species level, the “Carbohydrate Metabolism”, “Translation”, “Immune System” and the “Transcription” pathways were positively correlated with the abundance of *Lactobacillus* or *Lactobacillus reuteri*.

## Conclusion

Overall, our study demonstrated that obese mice exposed to chronic unpredictable mild stress suffered aggravation of relevant disease indicators, whereas gavage of *Lactobacillus reuteri* strain 8008 could alleviate depressive-like behavior and inflammatory responses as well as improve glucose and lipid metabolisms, intestinal barrier functions and intestinal bacteria distribution in obesity and depression comorbidity mice through regulating the gut microbiota. *Lactobacillus reuteri* strain 8008 may be a potential probiotic agent for improving symptoms associated with obesity and depression comorbidity disorders. Nevertheless, there are certain limitations to our study. The animal model we have used to mimic obesity and depression comorbidity disease still has certain limitations and it did not completely represent the complex features of the disease. In addition, the role of gut microbes plays in glucose and lipid metabolisms as well as insulin resistance still needs to be investigated in-depth.

## Data Availability

The datasets presented in this study can be found in online repositories. The names of the repository/repositories and accession number(s) can be found below: https://www.ncbi.nlm.nih.gov/bioproject/PRJNA933776.
