# Corrigendum: *Lactobacillus reuteri* strain 8008 attenuated the aggravation of depressive-like behavior induced by CUMS in high-fat diet-fed mice through regulating the gut microbiota

**DOI:** 10.3389/fphar.2023.1318323

**Published:** 2023-10-20

**Authors:** Canye Li, Zuanjun Su, Zhicong Chen, Jinming Cao, Xiufeng Liu, Feng Xu

**Affiliations:** ^1^ Fengxian Hospital, Southern Medical University, Shanghai, China; ^2^ Sixth People’s Hospital South Campus, Shanghai Jiaotong University, Shanghai, China

**Keywords:** chronic unpredictable mild stress, obesity, depression, *Lactobacillus reuteri*, mice

In the published article, there was an error in **Affiliation** 1. Instead of “School of Pharmaceutical Sciences, Southern Medical University, Guangzhou, China”, it should be “Fengxian Hospital, Southern Medical University, Shanghai, China”.

The authors apologize for this error and state that this does not change the scientific conclusions of the article in any way. The original article has been updated.

